# Leveraging medicare claims to reduce false positives from 87% to 12%, a two‐stage approach to dementia screening

**DOI:** 10.1002/alz70856_107487

**Published:** 2026-01-09

**Authors:** MacKenzie Tweardy, Keith J Yoder, Spencer Gerrol, Ché Lucero

**Affiliations:** ^1^ SPARK Neuro, Inc., New York, NY, USA

## Abstract

**Background:**

Some 40% ‐ 60% of dementia goes undiagnosed in the United States, creating missed opportunities and worsening outcomes for patients. Medicare recommends yearly cognitive screening for the elderly, but fewer than a quarter of those patients get cognitive assessments. Why might this be? Community screening for dementia with common pen‐and‐paper instruments yields mostly false positives (around 85%). Here, we demonstrate that these false positives can be dramatically reduced by first using a machine‐learning model trained on Medicare claims data to exclude individuals most likely to be healthy to focus assessments on those most likely to have undiagnosed dementia.

**Method:**

We analyzed a 5‐year span of Medicare claims data, reflecting 40 million claims from 1.9 million individuals. We extracted features by calculating the proportion of claims within a given time period that were related to a set of literature‐based diagnoses and risk factors, such as diabetes and hypertension. We then trained a gradient‐boosted decision tree (XGBoost) to detect undiagnosed dementia. To understand whether this model could be combined with a cognitive screener to reduce false positives, we evaluated its expected performance at detecting undiagnosed dementia in a hypothetical population of 100,000 older adults. We compared the false positive rate for detecting new dementia cases when a brief cognitive screener was used alone or applied only to those first flagged by the claims‐based model.

**Result:**

Using performance from a recent meta‐analysis of sensitivity (76%) and specificity (83%), and an assumed undiagnosed dementia prevalence of 4%, the Mini‐Cog resulted in a false positive rate of 84.3%. In contrast, administering the Mini‐Cog only to those individuals flagged by the claims‐based model, reduced the false positive rate to 12.7%. This approach decreased the total number of Mini‐Cog assessments from 100,000 to 2,924 while identifying 1,347 new dementia cases.

**Conclusion:**

Reducing false positives is key to making screening practical. We find that using a machine learning approach to claim‐analysis can identify patients who should be screened with a test like the Mini‐Cog. This creates a practical path to discovering and diagnosing dementia and connecting patients with appropriate care.

